# Evaluation of the Levels of Pain, Discomfort, Functional Impairments and Satisfaction With the Periodontally Accelerated Osteogenic Orthodontics (PAOO) When Leveling and Aligning Crowded Teeth: A Prospective Cohort Study

**DOI:** 10.7759/cureus.22623

**Published:** 2022-02-26

**Authors:** Hallaj I Alsino, Mohammad Y Hajeer, Issam Alkhouri, Rashad M.T. Murad, Tarek Z. Khattab

**Affiliations:** 1 Department of Orthodontics, University of Damascus, Faculty of Dentistry, Damascus, SYR; 2 Department of Oral and Maxillofacial Surgery, University of Damascus, Faculty of Dentistry, Damascus, SYR; 3 Department of Toxins and Pharmaceutics, University of Damascus, Faculty of Pharmacology, Damascus, SYR; 4 Department of Orthodontics, University of Hamah, Faculty of Dentistry, Hamah, SYR

**Keywords:** satisfaction, functional impairment, discomfort, pain, xenograft, piezosurgery, crowded lower anterior teeth, visual analog scale (vas), periodontally accelerated osteogenic orthodontics (paoo)

## Abstract

Background

Only a few studies have reported levels of pain, discomfort, functional impairments, and patients' satisfaction after undergoing periodontally accelerated osteogenic orthodontics (PAOO). Therefore, the objectives were to evaluate postoperative pain, discomfort, functional impairments, and satisfaction following this surgical intervention.

Methods

A prospective cohort study was undertaken at the Department of Orthodontics, University of Damascus Dental School, between August 2018 and November 2020. The sample consisted of 16 patients (4 males, 12 females, mean age: 21±3.05 years) with moderately crowded lower anterior teeth who underwent full-thickness vestibular flap elevation followed by cortical cuts using piezosurgery. Then a bovine xenograft was applied before reflecting the flap and suturing. Fixed orthodontic therapy was then initiated. Questionnaires were administered to assess the levels of pain, discomfort, and oral functional problems using a visual analog scale (VAS) at one day, seven days, 14 days, and 28 days after surgery. The level of satisfaction was also assessed at 28 days postoperatively. Friedman's test was employed, and Wilcoxon signed-rank tests were used for post-hoc tests with an adjusted alpha level.

Results

On the first day following the PAOO, the mean values of perceived pain, discomfort, swelling, mastication difficulties, swallowing problems, limitation in jaw movements were 80.00, 80.63, 68.13, 78.13, 55.00, and 64.38, respectively. These mean values dropped significantly in the following assessment times (P<0.001). At 28 days following the PAOO, the mean satisfaction score was 84.94±22.46. All patients mentioned that they took painkillers after the surgical intervention.

Conclusions

On the first day following surgery, patients perceived high levels of pain and discomfort, moderate to severe levels of swelling and chewing difficulties, and suffered from restricted jaw movement. These levels decreased significantly in the following assessment times. Patients' satisfaction with the PAOO procedure was high.

## Introduction

Pain complaints are a common condition during orthodontic treatment [1], which directly affects patient satisfaction [2]. Fear of pain is one of the primary reasons that patients fail to seek orthodontic care [3]. In addition to the long duration of orthodontic treatment, many patients may decline treatment because most conventional orthodontic treatments require more than one year to complete [4].

These considerations make orthodontic treatment of adults different and challenging as well as necessitate special concepts and procedures, such as the use of invisible appliances, shorter periods of treatment, the use of lighter forces, and more precise tooth movements [5]. The development of corticotomy-assisted orthodontic treatment (CAOT) has opened doors and offered solutions to many limitations in the orthodontic treatment of adults [6]. Surgical procedures to accelerate tooth movement have been commonly used [4], with results that may appear better in reducing the duration of orthodontic treatment [7].

Periodontal accelerated osteogenic orthodontics (PAOO) has recently gained popularity and acceptance because proponents of this technique claim that it is safe and effective in accelerating tooth movement as well as benefits compared to conventional orthodontic treatment [8]. Conversely, the introduction of a surgical phase to orthodontic therapy may prevent a patient from considering PAOO as a treatment option due to the aggressive nature of the procedure [9]. To the best of our knowledge, only two studies assessed postoperative pain levels [10,11]. Ma et al., in a cohort study involving 12 patients, assessed pain levels at two assessment time points (one week and two weeks postoperatively), but there was no assessment in the immediate stage following surgery, i.e. within the first three days [10]. The intensity of pain in the study of Muñoz et al. was interpreted according to the patients' need for and consumption of NSAIDs following surgery without resorting to their actual feelings of pain depending on patient-reported scales of perceived pain [11]. However, the previous two studies did not assess levels of acceptance, discomfort, functional impairment, and patient satisfaction with the surgical intervention. Therefore, the current cohort study evaluated pain, discomfort, swelling, difficulties of mastication, swallowing, jaws movement limitation, and patient satisfaction levels after the surgical intervention in PAOO patients. It was proposed that if there was evidence of high postoperative levels of pain, discomfort, or functional impairments, this could decrease the use of this technique by orthodontists and decrease patients' acceptance of such treatment. 

## Materials and methods

Study design and settings

A prospective cohort study was undertaken at the Department of Orthodontics, University of Damascus Dental School between the 29th of August 2018 and the 1st of November 2020. This study was registered at Clinical Trials.gov (NCT04728464). The Local Research Ethics Committee Approval was obtained (UDDS-480-01012018/SRC-2100) and was funded by the University of Damascus Dental School Postgraduate Research Budget (Ref no: 83004660112DEN).

Sample size estimation

The sample size was calculated using the Minitab® 18.1 software (Minitab LLC, State College, Pennsylvania, US) with an alpha level of 0.05, a power of 80%. The most important variable in the current study was pain perception. According to the study of Ma et al., the standard deviation of pain was 1.27 after one-week post-surgically assessed on a 10-mm VAS [10]. The least clinically significant difference requiring detection overtime was assumed 10 mm on a 100-mm VAS. Using paired t-test, the required sample size was 15 patients. However, with an assumed withdrawal percentage of 5%, the required sample size was increased to 16 patients.

Patients' recruitment and inclusion in the study

The patients were examined at the Orthodontics Department of the University of Damascus Dental School. The sample was composed of patients who were planned to be treated in conjunction with the PAOO procedure. Initially, 50 patients were screened and only 28 of these patients met the inclusion criteria. The research-related information sheet was presented to the patients and three patients refused the surgical intervention. Consent forms were obtained from those who agreed to participate. Therefore, 16 patients out of the remaining 25 patients were randomly selected to enter this cohort study.

The inclusion criteria were: (1) adult healthy patients from both sexes within an age range 18-28 years; (2) an absence of previous orthodontic treatment; (3) class I malocclusion; (4) moderate crowded lower anterior teeth; (5) good oral hygiene and healthy periodontium which was evaluated clinically: probing depth ≤3mm, no radiograph evidence of bone loss, plaque and gingival index <1 [12].

Exclusion criteria were: (1) medical conditions that affect tooth movement; (2) medical, social, and psychological contraindications to oral surgery; (3) the presence of primary teeth in the mandibular arch; (4) missing permanent mandibular teeth (except third molars); (5) patients had previous orthodontic treatments; (6) poor oral hygiene or concurrent periodontal disease: probing depth ≥4 mm, radiographic evidence of bone loss, gingival index > 1, plaque index > 1 [12].

The surgical procedure: PAOO

**Figure 1:**
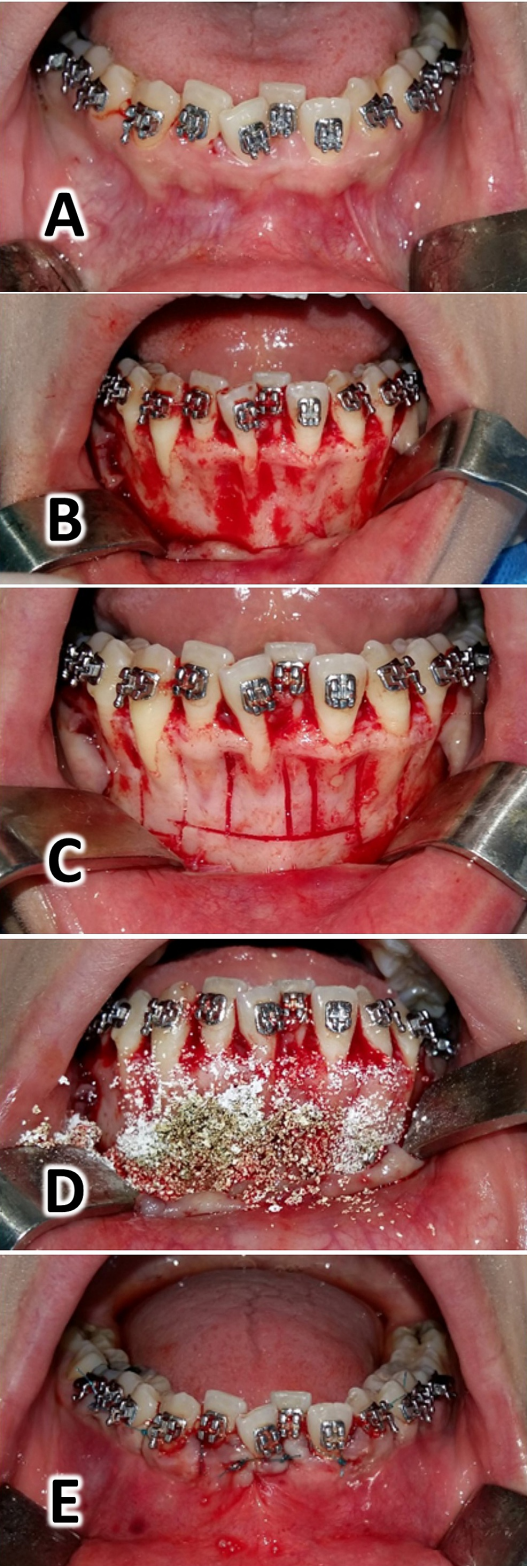
Surgical intervention procedures: Local anesthesia (lidocaine HCL 2% - epinephrine 1:80000) (A). The full-thickness flap was raised labially only (B). The selective cortical cut was performed according to vertical cutting lines between the roots and ended with a horizontal cutting line using a piezosurgery (C). The xenograft (Bone-D®, MedPark Co, Busan, Korea, the particle size of 0.2 mm–1.0 mm) was placed (D). The wound was sutured using a 2 metric nylon 3/0 suture on a reverse cutter needle (Shandong, China) (E).

Surgery was performed at the Department of Oral and Maxillofacial Surgery at the Faculty of Dentistry at Damascus University. A postgraduate student at the Department of Oral and Maxillofacial Surgery performed all surgical interventions under the supervision of one of the co-authors (IA). All surgical procedures were conducted according to the method suggested by Bahammam [13] (Figure 1). The full-thickness flap was raised labially only under local anesthesia, from the distal surface of the lower right canine to the distal surface of the lower left canine. The exposed alveolar bone was washed with saline and a selective cortical cut was performed according to vertical cutting lines between the roots and ended with a horizontal cutting line using a piezosurgery and then the xenograft (Bone-D®, MedPark Co, Busan, Korea; particle size of 0.2 mm-1.0 mm) was placed, then the wound was sutured.

Following surgery, Amoxicillin 500 mg tab was prescribed three times daily for one week; paracetamol 500 mg if necessary and chlorhexidine gluconate 0.12% twice a day. The patient was directed to fill out a pain and discomfort questionnaire before taking the analgesic drug and after anesthesia has worn off. Then a schedule was set up to review the patient after 1, 7, 14, and 28 days to continue treatment. Sutures were removed after seven days.

Orthodontic procedures

On the 14th day of the surgical procedure, all participants underwent orthodontic fixed appliance treatment with an MBT prescription using a 0.022-inch bracket-slot (Votion™, Ortho Technology®, Florida, USA). The first archwire was applied on the same day with a size of 0.012-in. NiTi. Follow-up visits were conducted every two weeks employing the following sequence of archwires: 0.014-in. NiTi, 0.016-in. NiTi, 0.016 X 0.022-in. NiTi, 0.017 X 0.025-in. NiTi, 0.019 X 0.025-in. NiTi and finally 0.019 X 0.025-in. stainless steel [14]. Change in archwires was performed every two weeks when it was felt that an improvement occurred in teeth positions and there was a possibility of inserting the next archwire without exerting excessive force on the engaged teeth [14].

Outcome measures: pain, discomfort and functional impairments questionnaire

**Figure 2:**
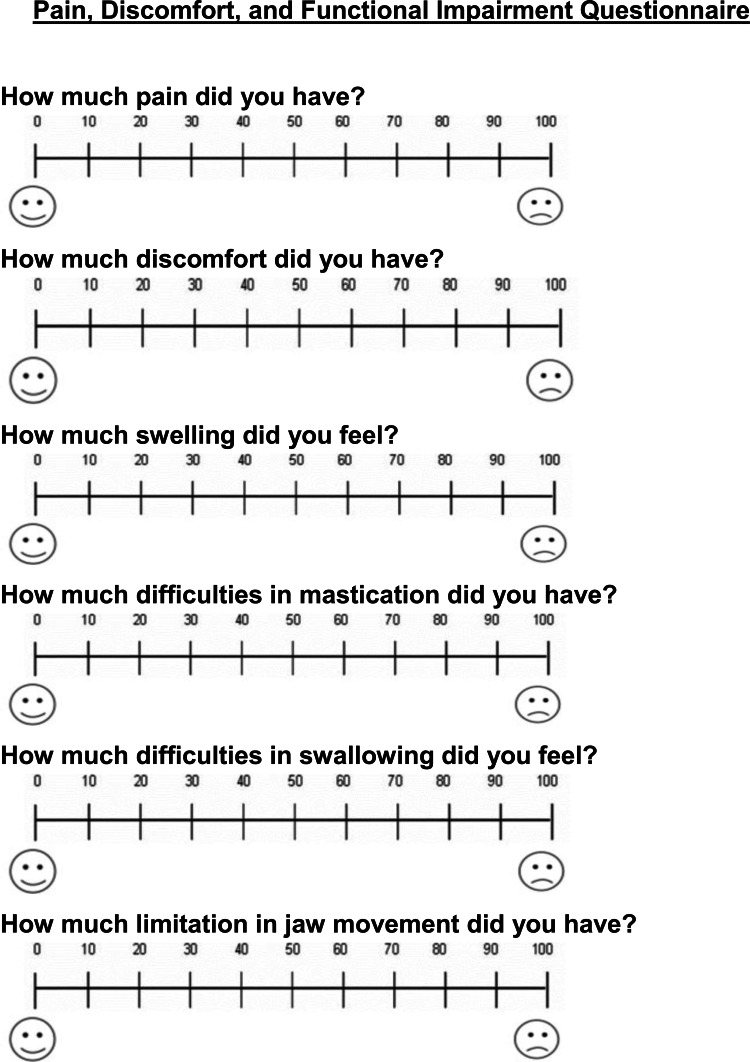
This questionnaire was administered at one day, seven days, and 14 days following the surgical intervention.

**Figure 3:**
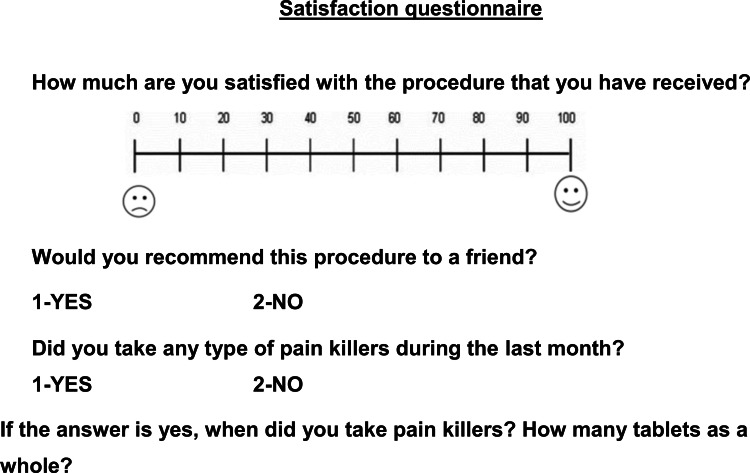
The questionnaire administered to patients at 28 days following the surgical intervention

The outcome measures for patients treated with PAOO included levels of pain, discomfort, and swelling, difficulties of mastication, swallowing, and jaws movement limitation. The questionnaire was given to patients one day following surgery and at the following assessment times: seven days, 14 days, and 28 days post-surgically. All patients were instructed to mark their responses on a visual analog scale (VAS). This questionnaire was first suggested by Sergl and Zentner [15], modified by Idris et al. [16], and was further modified by us to conform to this cohort study. A line of 100-mm length was used with the left side representing no pain, discomfort, swelling, difficulties of mastication, swallowing, or jaws movement limitation (i.e., score = 0) and the right side representing the worst pain, highest levels of discomfort, swelling, difficulties of mastication, swallowing and jaws movement limitation (i.e., score = 100). Each patient was asked to put a vertical mark on the line at a point that best represented the perceived levels of the aforementioned variables (Figure 2). At 28 days following surgery (i.e., the last assessment time), the patient's satisfaction with the surgical intervention accompanying the PAOO was assessed by guiding all patients to rate their level of satisfaction on a visual analog scale (VAS). A line of length 100 mm was used with the left side representing the worst satisfaction from the therapeutic intervention (i.e., score = 0) and the right side representing the best satisfaction from the therapeutic interaction (i.e., score = 100), and also knew whether the patient would recommend this intervention to their friends. Patients were also asked to record any consumption of analgesics during the first week following surgery (Figure 3). Accordingly, pain severity was classified depending on the number of painkillers being taken during the first week to the following categories: mild pain (less than four tablets), moderate pain (four to eight tablets), and severe pain (more than eight tablets).

Statistical analysis

Using Minitab® Version 18.1, Anderson-Darling Normality tests showed an abnormal distribution of the collected data, so non-parametric tests were used. Friedman's test was used to detect changes over time with an alpha level set at 5%. Wilcoxon signed-rank tests were employed for pairwise comparisons. Bonferroni's correction was employed due to multiplicity. All statistical analyses were performed using SPSS® Statisics version 24.0 (IBM Corp., Armonk, NY, USA).

## Results

All patients had Class I malocclusion with moderate crowding on the lower anterior teeth (i.e., 5.06 ± 0.87 millimeters of a tooth size-arch length discrepancy). The sample consisted of 4 males and 12 females. Patients' age ranged from 18 to 28 years with an average of 21±3.05. Descriptive and inferential statistics of the collected scores at the different assessment times are given in Tables 1-2.

**Table 1:** Descriptive and inferential statistics of the collected scores at the different assessment times using a visual analog scale (n=16) * Friedman's test; **significant at P<0.05; SD: Standard deviation; Min: Minimum, Max: Maximum; T1: one day following surgery; T2: 7 days postoperatively; T3: 14 days postoperatively, T4: 28 days post-operatively.

Variable	Time	Mean	SD	Min	Max	Median	P-value*
Pain	T1	80.00	19.66	40	100	85.00	<0.001**
	T2	45.00	28.28	0	100	50.00	
	T3	11.88	17.97	0	50	0.00	
	T4	1.25	3.41	0	10	0.00	
Discomfort	T1	80.63	16.91	60	100	80.00	<0.001**
	T2	46.25	25.26	20	100	40.00	
	T3	19.38	19.13	0	60	15.00	
	T4	6.88	8.73	0	20	0.00	
Swelling	T1	68.13	17.59	30	90	70.00	<0.001**
	T2	36.88	21.51	10	90	35.00	
	T3	14.38	13.64	0	40	10.00	
	T4	1.25	3.41	0	10	0.00	
Mastication	T1	78.13	18.69	40	100	80.00	<0.001**
	T2	38.13	28.33	0	90	40.00	
	T3	20.00	18.61	0	60	20.00	
	T4	44.38	20.96	20	90	40.00	
Swallowing	T1	55.00	23.66	20	100	60.00	<0.001**
	T2	20.63	16.91	0	50	20.00	
	T3	6.25	8.85	0	20	0.00	
	T4	0.63	2.50	0	10	0.00	
Limitation	T1	64.38	23.37	30	100	60.00	<0.001**
	T2	25.00	20.97	0	70	25.00	
	T3	4.38	6.29	0	20	0.00	
	T4	0.31	1.25	0	5	0.00	

**Table 2:** P-values of pairwise comparisons between assessment times regarding the evaluated variables (n=16) The Wilcoxon signed-ranks tests were employed for pairwise comparisons, with the Bonferroni's adjustment of alpha level (i.e., 0.05/6 = 0.008). * P < 0.008 was considered statistically significant. T1: one day following surgery; T2: 7 days postoperatively; T3: 14 days postoperatively, T4: 28 days post-operatively.

Time intervals	Pain	Discomfort	Swelling	Mastication	Swallowing	Limitation
T1 – T2	0.001*	0.001*	0.001*	<0.001*	<0.001*	<0.001*
T1 – T3	<0.001*	<0.001*	<0.001*	<0.001*	<0.001*	<0.001*
T1 – T4	<0.001*	<0.001*	<0.001*	0.001*	<0.001*	<0.001*
T2 – T3	0.001*	<0.001*	0.001*	0.003*	0.002*	0.002*
T2 – T4	0.001*	<0.001*	<0.001*	0.330	0.003*	0.002*
T3 – T4	0.026	0.007*	0.005*	0.001*	0.024	0.020

Regarding pain assessment, patients' responses ranged from 40 to 100 mm on the VAS scale on the first postoperative day and the mean pain value was high (80±19.66). Then, the pain level decreased after seven days, 14 days, and 28 days (45±28.28, 11.88±17.97, 1.25±3.41, respectively). This decrease was statistically significant between assessment times (P<0.001) except for the T3-T4 comparison which was not statistically significant (P=0.026).

Patients' responses regarding their feelings of discomfort ranged from 60 to 100 mm on the VAS scale on the first postoperative day and the mean discomfort value was high ( =80.63±16.91). Then the discomfort level decreased after seven days, 14 days, and 28 days (46.25±25.26, 19.38±19.13, 6.88±8.73, respectively). This decrease was statistically significant between assessment times (P<0.001).

When the level of observed swelling was evaluated, the mean value of swelling ranged from moderate to high on the first day after surgery (68.13±17.59). Then, the perception of swelling decreased after seven days, 14 days, and 28 days (36.88±21.51, 14.38±13.64, 1.25±3.41, respectively). This decrease was statistically significant between assessment times (P<0.001).

The level of perceived difficulties in mastication ranged from moderate to high on the first postoperative day (78.13±18.69), then the difficulties in mastication level decreased after seven days, 14 days, and 28 days (38.13±28.33, 20.00±18.61, 44.38±20.96, respectively). This decrease was statistically significant between assessment times (P<0.001) except for the T2-T4 comparison which was not statistically significant (P=0.330).

Difficulty swallowing was noted on the first postoperative day and the mean difficulties in swallowing value were moderate (55.00±23.66). The difficulties in swallowing level decreased after seven days, 14 days, and 28 days (20.63±16.91, 6.25±8.85, 0.63±2.50, respectively). This decrease was statistically significant between assessment times (P<0.001) except for the T3-T4 comparison which was not statistically significant (P=0.024).

The mean limitation in jaws movement ranged from moderate to high on the first postoperative day (64.38±23.37). The limitation in jaws movement level decreased after seven days, 14 days, and 28 days (25.00±20.97, 4.38±6.29, 0.31±1.25, respectively). This decrease was statistically significant between assessment times (P<0.001) except for the T3-T4 comparison which was not statistically significant (P=0.020).

After the PAOO intervention, the patients' satisfaction with the intervention was high (84.94±22.46). Also, 75% of the patients recommended this procedure to their friends. All patients took painkillers after the surgical intervention, eight patients were classified as having mild pain (50%), four patients had moderate pain (25%), and four patients had severe pain (25%).

## Discussion

This is the first cohort study that assessed pain, acceptance, and discomfort levels in patients treated with PAOO by administering questionnaires at one, seven, 14, and 28 days after surgery. The VAS was used as a tool to measure the level of pain after the surgical procedure. The VAS is more commonly used in oral and maxillofacial surgery (OMFS) research because it is more reliable, valid, sensitive, and appropriate [17]. This is in agreement with previous studies that used the VAS to measure the level of pain after using piezosurgery to accelerate tooth movement [10,18].

The current findings showed that the mean pain value was high (80±19.66) on the first postoperative day. The pain was severe on the first day due to trauma and edema as a result of the surgery. Also, 25% of patients had severe pain and took painkillers heavily during the first week following surgery; whereas a clinical trial by Chandra et al. showed that the pain level was moderate immediately after surgery in the PAOO group compared to the traditional corticotomy group without any significant difference between them [19]. It should be noted that Chandra et al. assessed the level of pain immediately after surgery and this did not reflect the actual pain level due to the persistence of the local anesthetic effect. In a cohort study of patients with various malocclusion who underwent a PAOO procedure, Muñoz et al. classified pain as being 'severe' when their patients consumed analgesics for more than six days following the surgical intervention [11]. However, Muñoz's assumption of pain severity when analgesics are taken for more than six days does not reflect the actual severity of acute pain when it occurs within the first two days following surgery. Therefore, direct comparisons with their results are not straightforward.

Additionally, the results of the current study agreed with the results of Al-Naoum et al. who conducted a clinical trial evaluating canine retraction after traditional corticotomy with flap elevation. They found that 23.33% of their patients reported severe daytime pain during the first postoperative day [20].

The level of pain decreased statistically significantly after one week of the surgical intervention. The results of this study were also in agreement with those of a cohort study accomplished by Ma et al. who included class II and class III patients treated in conjunction with a PAOO procedure. Patients in that study experienced moderate pain in the first week following surgery [10]. However, Ma et al. did not capture patients' perception of pain in the immediate post-surgical period, since their first assessment was at the first week following PAOO.

In the present study, the level of discomfort was severe on the first day after surgery but decreased significantly to a moderate level on the seventh day following the surgical intervention. Al-Naoum et al. in their clinical trial that evaluated canine retraction after traditional corticotomy reported that 10% of patients experienced severe discomfort on the first day after the surgical intervention, using a four-point Likert scale. Subsequently, no patient experienced any discomfort after a week of the surgical intervention [20]. The difference in the results of the current study with those of Al-Naoum et al. could be explained by the difference in the method and location of the surgical intervention.

The difficulty in swallowing was moderate on the first day after surgery (x ®=55.00±23.66). This can be explained by limitations in jaw movement following surgical intervention. Subsequently, the difficulty in swallowing reduced significantly after one week of surgery. There are no studies that have evaluated swallowing difficulties in patients undergoing PAOO procedures, therefore, direct comparison of the current results with other studies is not possible. However, in a clinical trial evaluating the effectiveness of flapless corticotomy in leveling and alignment of severely crowded lower anterior teeth, the level of difficulty in swallowing was found mild on the first day after surgery. The difference can be attributed to the conservative nature of the surgical intervention in the study by Gibreal et al [14].

Difficulty in chewing was reported to range from moderate to severe on the first day after the intervention (x ®=78.13±18.69) due to postoperative pain, edema, and swelling. Chewing improved after Day 7 and Day 14 of intervention. The level of chewing difficulty increased again on Day 28 after surgery due to the orthodontic tooth movement following the insertion of archwires on the 14th postoperative day. Unfortunately, the results of chewing impairments in the current work cannot be compared with others' findings due to the absence of similar studies.

The swelling occurred significantly and was reported as moderate to severe (68.13±17.59) on the first postoperative day due to the surgical trauma. The swelling was visible on the patients' frontal view and was located in the chin and sub chin area. Patients were not prescribed NSAIDs after the surgical intervention, and they were instructed to place cold bandages on the chin area during the day of surgery. Subsequently, the swelling decreased significantly to a mild level in the first week following the intervention. This goes in line with the results of Ma et al., who included 12 patients treated with PAOO and showed severe swelling in the first week after surgery [10].

Limitation in jaws movement occurred significantly and was reported as moderate to severe (64.38±23.37) on the first postoperative day. Subsequently, the limitation in jaw movement decreased and was reported as mild to moderate on the seventh postoperative day (25.00±20.97). The results of the current study agreed with those of Ma et al., who recorded a mild to moderate incidence of trismus in patients at the first week after surgery [10].

Patient satisfaction with the therapeutic interaction was high (84.94±22.46) and this is similar to the results of Chackartchi et al., where the satisfaction score was 9.78 on the VAS scale [18]. Moreover, 75% of patients said they would recommend the PAOO procedure to their friends.

Limitations

The surgical intervention was confined to the lower jaw only. This does not probably reflect the actual level of postoperative pain, discomfort, or functional impairments if the study involved both jaws in this operation. In addition, the surgical intervention was performed on the buccal side of the alveolar bone only and not on both sides as recommended in the primary work of Wilcko et al [8]. The current study did not take into account the long-term effects of the PAOO procedure.

Generalizability

The possibility of generalizing the results of the current study may be limited, as it included patients with specific malocclusion with a specific age group. More controlled cohort studies of different cases of malocclusion are preferred to generalize the findings.

## Conclusions

Orthodontic treatment of crowded lower anterior teeth in conjunction with the PAOO procedure was associated with a high level of pain and discomfort, moderate to severe levels of swelling, difficulty in chewing, and restricted jaw movement. Levels of pain, discomfort, and functional impairments reduced significantly after seven, 14, and 28 days following surgery (p<0.001). Patient satisfaction was high with the PAOO procedure and 75% of patients agreed to recommend this therapeutic intervention to their friends.

## References

[1] Kazancı F, Aydoğan C, Alkan Ö (2016). Patients' and parents' concerns and decisions about orthodontic treatment. Korean J Orthod.

[2] Al-Omiri MK, Abu Alhaija ES (2006). Factors affecting patient satisfaction after orthodontic treatment. Angle Orthod.

[3] Oliver RG, Knapman YM (1985). Attitudes to orthodontic treatment. Br J Orthod.

[4] Alghamdi AS (2010). Corticotomy facilitated orthodontics: Review of a technique. Saudi Dent J.

[5] Khlef HN, Hajeer MY, Ajaj MA, Heshmeh O (2019). En-masse retraction of upper anterior teeth in adult patients with maxillary or bimaxillary dentoalveolar protrusion: a systematic review and meta-analysis. J Contemp Dent Pract.

[6] Amit G, Jps K, Pankaj B, Suchinder S, Parul B (2012). Periodontally accelerated osteogenic orthodontics (PAOO) - a review. J Clin Exp Dent.

[7] Alfawal AM, Hajeer MY, Ajaj MA, Hamadah O, Brad B (2016). Effectiveness of minimally invasive surgical procedures in the acceleration of tooth movement: a systematic review and meta-analysis. Prog Orthod.

[8] Wilcko WM, Wilcko T, Bouquot JE, Ferguson DJ (2001). Rapid orthodontics with alveolar reshaping: two case reports of decrowding. Int J Periodontics Restorative Dent.

[9] Murphy KG, Wilcko MT, Wilcko WM, Ferguson DJ (2009). Periodontal accelerated osteogenic orthodontics: a description of the surgical technique. J Oral Maxillofac Surg.

[10] Ma Z, Zheng J, Yang C, Xie Q, Liu X, Abdelrehem A (2018). A new modified bone grafting technique for periodontally accelerated osteogenic orthodontics. Medicine (Baltimore).

[11] Munoz F, Jiménez C, Espinoza D, Vervelle A, Beugnet J, Haidar Z (2016). Use of leukocyte and platelet-rich fibrin (L-PRF) in periodontally accelerated osteogenic orthodontics (PAOO): Clinical effects on edema and pain. J Clin Exp Dent.

[12] Silness J, Löe H (1964). Periodontal disease in pregnancy II. Correlation between oral hygiene and periodontal condition. Acta Odontol Scand.

[13] Bahammam MA (2016). Effectiveness of bovine-derived xenograft versus bioactive glass with periodontally accelerated osteogenic orthodontics in adults: a randomized, controlled clinical trial. BMC Oral Health.

[14] Gibreal O, Hajeer MY, Brad B (2019). Evaluation of the levels of pain and discomfort of piezocision-assisted flapless corticotomy when treating severely crowded lower anterior teeth: a single-center, randomized controlled clinical trial. BMC Oral Health.

[15] Sergl HG, Zentner A (1998). A comparative assessment of acceptance of different types of functional appliances. Eur J Orthod.

[16] Idris G, Hajeer MY, Al-Jundi A (2012). Acceptance and discomfort in growing patients during treatment with two functional appliances: a randomised controlled trial. Eur J Paediatr Dent.

[17] Sirintawat N, Sawang K, Chaiyasamut T, Wongsirichat N (2017). Pain measurement in oral and maxillofacial surgery. J Dent Anesth Pain Med.

[18] Chackartchi T, Barkana I, Klinger A (2017). Alveolar bone morphology following periodontally accelerated osteogenic orthodontics: a clinical and radiographic analysis. Int J Periodontics Restorative Dent.

[19] Chandra RV, Rachala MR, Madhavi K, Kambalyal P, Reddy AA, Ali MH (2019). Periodontally accelerated osteogenic orthodontics combined with recombinant human bone morphogenetic protein-2: An outcome assessment. J Indian Soc Periodontol.

[20] Al-Naoum F, Hajeer MY, Al-Jundi A (2014). Does alveolar corticotomy accelerate orthodontic tooth movement when retracting upper canines? A split-mouth design randomized controlled trial. J Oral Maxillofac Surg.

